# Supplementary material for: The relationship between protein domains and homopeptides in the Plasmodium falciparum proteome
Source: PeerJ. 2020 Oct 2;8:e9940. doi: 10.7717/peerj.9940 (PMC7534687; doi:10.7717/peerj.9940)
Supplement: Supplemental Information 1 [file peerj-08-9940-s001.docx]

#

#

# Total # of K-homopeptide-enriched-domains = 50

# Total # of R-homopeptide-enriched-domains = 9

# Total # of N-homopeptide-enriched-domains = 87

# Total # of D-homopeptide-enriched-domains = 22

# Total # of A-homopeptide-enriched-domains = 8

# Total # of L-homopeptide-enriched-domains = 28

# Total # of I-homopeptide-enriched-domains = 19

# Total # of G-homopeptide-enriched-domains = 25

# Total # of (>=10 residues) N-homopeptide-enriched-domains = 29

#

# The top ten of these homopeptide-enrichments for protein domains

# are listed below. Domains in multiple lists are asterisked.

#

# The columns are:

# identifier; total number of residue-type homopeptides in domains;

# total number in this domain; total number of residues in domains;

# total number of residues in this domain; ln(p-value) for enrichment (used for

# sorting); P-value for enrichment; Name of the domain (asterisked

# if it appears in multiple top ten lists)

#

#

#

K-homopeptide-enriched

----------------------

PF02009 5036 755 808565 49394 -258.652 4.66483e-113 *Rifin

PF03011 5036 227 808565 16464 -61.602 1.76433e-27 PfEMP DBL domain

PF03416 5036 39 808565 737 -52.6973 1.29971e-23 *Peptidase family C54

PF06978 5036 19 808565 141 -43.8706 8.85635e-20 *Ribonucleases P/MRP protein subunit POP1

PF04502 5036 27 808565 696 -29.696 1.26821e-13 Family of unknown function (DUF572)

PF05285 5036 21 808565 423 -28.0849 6.35149e-13 *SDA1

PF09716 5036 35 808565 1443 -24.559 2.15848e-11 Malarial early transcribed membrane protein (ETRAMP)

PF04712 5036 23 808565 632 -24.3353 2.69965e-11 Radial spokehead-like protein

PF13019 5036 9 808565 64 -22.0191 2.73683e-10 Silencing defective 2 N-terminal ubiquitin domain

PF14713 5036 13 808565 189 -21.9663 2.88506e-10 Domain of unknown function (DUF4464)

R-homopeptide-enriched

----------------------

PF05424 454 224 808565 30888 -429.648 2.54849e-187 Duffy binding domain

PF17410 454 78 808565 8693 -152.663 5.00619e-67 *Subtelomeric Variable Open Reading frame

PF08648 454 15 808565 267 -57.1354 1.53601e-25 U4/U6.U5 small nuclear ribonucleoproteins

PF00226 454 13 808565 2706 -18.788 6.92582e-09 DnaJ domain

PF06978 454 4 808565 141 -13.4551 1.43389e-06 *Ribonucleases P/MRP protein subunit POP1

PF17144 454 4 808565 164 -12.8574 2.60667e-06 Ribosomal large subunit proteins 60S L5, and 50S L18

D-homopeptide-enriched

----------------------

PF05285 881 33 808565 423 -112.913 9.17446e-50 *SDA1

PF02906 881 31 808565 368 -108.61 6.77824e-48 Iron only hydrogenase large subunit, C-terminal domain

PF04998 881 36 808565 2942 -57.7671 8.16737e-26 *RNA polymerase Rpb1, domain 5

PF05470 881 21 808565 711 -52.014 2.57404e-23 Eukaryotic translation initiation factor 3 subunit 8 N-terminus

PF01214 881 15 808565 379 -41.9561 6.00738e-19 Casein kinase II regulatory subunit

PF15445 881 84 808565 29629 -33.3492 3.28572e-15 acidic terminal segments, variant surface antigen of PfEMP1

PF04130 881 22 808565 2889 -26.6408 2.6918e-12 Gamma tubulin complex component C-terminal

PF01237 881 12 808565 575 -26.3946 3.4432e-12 Oxysterol-binding protein

PF00271 881 30 808565 6998 -21.6463 3.97303e-10 Helicase conserved C-terminal domain

PF06705 881 9 808565 455 -19.7187 2.73062e-09 SF-assemblin/beta giardin

N-homopeptide-enriched

----------------------

PF04801 3941 79 808565 685 -182.416 5.99724e-80 Sin-like protein conserved region

PF02383 3941 94 808565 1773 -145.18 8.89698e-64 SacI homology domain

PF04998 3941 78 808565 2942 -72.7409 2.56459e-32 *RNA polymerase Rpb1, domain 5

PF03635 3941 45 808565 982 -64.4678 1.00457e-28 Vacuolar protein sorting-associated protein 35

PF03416 3941 35 808565 737 -51.7515 3.34667e-23 *Peptidase family C54

PF12752 3941 17 808565 83 -51.0047 7.06204e-23 SUZ domain

PF00995 3941 60 808565 2584 -49.9356 2.05703e-22 Sec1 family

PF04053 3941 36 808565 877 -48.3848 9.69946e-22 Coatomer WD associated region

PF02940 3941 25 808565 334 -48.3182 1.03678e-21 mRNA capping enzyme, beta chain

PF00443 3941 72 808565 3928 -46.2755 7.99464e-21 Ubiquitin carboxyl-terminal hydrolase

A-homopeptide-enriched

----------------------

PF02009 723 466 808565 49394 -853.764 0 *Rifin

PF17410 723 37 808565 8693 -31.8559 1.46271e-14 *Subtelomeric Variable Open Reading frame

PF03297 723 6 808565 100 -21.3229 5.49024e-10 S25 ribosomal protein

PF00012 723 15 808565 3489 -14.0589 7.83961e-07 Hsp70 protein Hsp70 protein

PF07992 723 12 808565 2572 -12.3663 4.25967e-06 *Pyridine nucleotide-disulphide oxidoreductase

PF02866 723 6 808565 482 -12.1037 5.5392e-06 lactate/malate dehydrogenase, alpha/beta C-terminal domain

PF00493 723 12 808565 2894 -11.2322 1.32407e-05 MCM P-loop domain

PF12777 723 9 808565 1715 -10.5335 2.66293e-05 Microtubule-binding stalk of dynein motor

L-homopeptide-enriched

----------------------

PF06728 1676 20 808565 430 -46.04 1.01176e-20 GPI transamidase subunit PIG-U

PF00566 1676 25 808565 2092 -25.8819 5.74954e-12 Rab-GTPase-TBC domain

PF07774 1676 10 808565 186 -25.2715 1.05857e-11 ER membrane protein complex subunit 1, C-terminal

PF04118 1676 10 808565 250 -22.3826 1.9027e-10 Dopey, N-terminal

PF12796 1676 18 808565 1540 -18.8214 6.69848e-09 Ankyrin repeats

PF00690 1676 7 808565 137 -17.7742 1.90885e-08 Cation transporter/ATPase, N-terminus

PF02516 1676 10 808565 415 -17.5818 2.31375e-08 Oligosaccharyl transferase STT3 subunit

PF04054 1676 12 808565 728 -16.656 5.83947e-08 CCR4-Not complex component, Not1

PF03203 1676 6 808565 114 -15.6012 1.67687e-07 MerC mercury resistance protein

PF00005 1676 25 808565 3629 -15.2026 2.49794e-07 ABC transporter

I-homopeptide-enriched

----------------------

PF00270 1527 69 808565 9538 -45.7016 1.41922e-20 DEAD/DEAH box helicase

PF14360 1527 14 808565 215 -38.6783 1.59312e-17 PAP2 superfamily C-terminal

PF00957 1527 15 808565 447 -31.5554 1.97547e-14 Synaptobrevin

PF02009 1527 147 808565 49394 -17.5513 2.38536e-08 *Rifin

PF04791 1527 9 808565 352 -17.2481 3.23022e-08 LMBR1-like membrane protein

PF06432 1527 7 808565 270 -13.827 9.88564e-07 Phosphatidylinositol N-acetylglucosaminyltransferase

PF06294 1527 6 808565 186 -13.2867 1.69685e-06 CH-like domain in sperm protein

PF00510 1527 6 808565 192 -13.105 2.03509e-06 Cytochrome c oxidase subunit III

PF05185 1527 6 808565 192 -13.105 2.03509e-06 PRMT5 arginine-N-methyltransferase

PF00071 1527 16 808565 2046 -12.9843 2.29601e-06 Ras family

G-homopeptide-enriched

----------------------

PF02009 579 123 808565 49394 -76.431 6.40397e-34 *Rifin

PF07992 579 24 808565 2572 -42.4703 3.59255e-19 *Pyridine nucleotide-disulphide oxidoreductase

PF00501 579 33 808565 6924 -37.9005 3.4675e-17 AMP-binding enzyme

PF00091 579 15 808565 1338 -29.7265 1.23017e-13 Tubulin/FtsZ family, GTPase domain

PF00380 579 9 808565 378 -24.9819 1.41418e-11 Ribosomal protein S9/S16

PF03947 579 9 808565 385 -24.8199 1.66282e-11 Ribosomal Proteins L2, C-terminal domain

PF00162 579 9 808565 397 -24.5493 2.17962e-11 Phosphoglycerate kinase

PF00181 579 6 808565 238 -17.4499 2.64003e-08 Ribosomal Proteins L2, RNA binding domain

PF00118 579 18 808565 5010 -17.1738 3.47958e-08 TCP-1/cpn60 chaperonin family

PF02812 579 6 808565 258 -16.975 4.24483e-08 Glu/Leu/Phe/Val dehydrogenase, dimerisation domain

(>=10 residues)N-homopeptide-enriched

----------------------------

PF02383 485 55 808565 1773 -169.884 1.6601e-74 *SacI homology domain

PF04801 485 29 808565 685 -98.8759 1.14486e-43 *Sin-like protein conserved region

PF10408 485 21 808565 839 -60.9611 3.34927e-27 Ubiquitin elongating factor core

PF03901 485 18 808565 602 -55.6437 6.827e-25 Alg9-like mannosyltransferase family

PF00270 485 40 808565 9538 -47.5484 2.2386e-21 *DEAD/DEAH box

PF00501 485 34 808565 6924 -45.2944 2.13249e-20 * AMP-binding enzyme

PF03635 485 17 808565 982 -43.4846 1.30283e-19 * Vacuolar protein sorting-associated protein 35

PF14413 485 10 808565 120 -41.9615 5.97543e-19 Thg1 C terminal domain

PF16837 485 10 808565 124 -41.6231 8.38101e-19 Pre-mRNA-splicing factor SF3A3, of SF3a complex, Prp9

PF06544 485 11 808565 306 -36.6194 1.24849e-16 Protein of unknown function (DUF1115)
