# Supplementary material for: The relationship between protein domains and homopeptides in the Plasmodium falciparum proteome
Source: PeerJ. 2020 Oct 2;8:e9940. doi: 10.7717/peerj.9940 (PMC7534687; doi:10.7717/peerj.9940)
Supplement: Supplemental Information 2 [file peerj-08-9940-s002.docx]

homopeptides in pfam domains enrichment/depletion for Gene Ontology

--------------------------------------------------------------------

$1 = GO-term ID

$2 = total length of homopeptides in domains

$3 = length of homopeptides in domains with the GO term

$4 = total domain-length

$5 = length of domains with the GO term

$6 = ln(p-value)

$7 = p-value (threshold: 0.05/685=7.299e-5)

--------------------------------------------------------------------

homopeptide-enriched GO terms

--------------------------------------------------------------------

GO:0042578 19277 124 808565 1773 -57.1484 1.51619e-25 GO:phosphoric ester hydrolase activity

GO:0016192 19277 429 808565 10740 -54.6091 1.92113e-24 GO:vesicle-mediated transport

GO:0001534 19277 63 808565 632 -47.106 3.48423e-21 GO:radial spoke

GO:0060271 19277 63 808565 632 -47.106 3.48423e-21 GO:cilium assembly

GO:0060294 19277 63 808565 632 -47.106 3.48423e-21 GO:cilium movement involved in cell motility

GO:0030906 19277 75 808565 982 -40.252 3.30193e-18 GO:retromer, cargo-selective complex

GO:0042147 19277 75 808565 982 -40.252 3.30193e-18 GO:retrograde transport, endosome to Golgi

GO:0006338 19277 34 808565 274 -32.7171 6.18245e-15 GO:chromatin remodeling

GO:0043486 19277 34 808565 274 -32.7171 6.18245e-15 GO:histone exchange

GO:0005634 19277 288 808565 7569 -31.9116 1.38341e-14 GO:nucleus

GO:0000226 19277 138 808565 2889 -31.1147 3.06934e-14 GO:microtubule cytoskeleton organization

GO:0000922 19277 138 808565 2889 -31.1147 3.06934e-14 GO:spindle pole

GO:0005815 19277 138 808565 2889 -31.1147 3.06934e-14 GO:microtubule organizing center

GO:0007020 19277 138 808565 2889 -31.1147 3.06934e-14 GO:microtubule nucleation

GO:0043015 19277 138 808565 2889 -31.1147 3.06934e-14 GO:gamma-tubulin binding

GO:0004651 19277 36 808565 334 -30.1947 7.70225e-14 GO:polynucleotide 5'-phosphatase activity

GO:0005198 19277 98 808565 1813 -29.5629 1.44879e-13 GO:structural molecule activity

GO:0006886 19277 355 808565 10045 -29.0945 2.31427e-13 GO:intracellular protein transport

GO:0003899 19277 435 808565 12974 -27.6263 1.00476e-12 GO:DNA-directed 5'-3' RNA polymerase activity

GO:0006904 19277 122 808565 2584 -27.0853 1.72588e-12 GO:vesicle docking involved in exocytosis

GO:0015031 19277 91 808565 1706 -26.9364 2.00287e-12 GO:protein transport

GO:0033179 19277 64 808565 1006 -26.6341 2.70995e-12 GO:proton-transporting V-type ATPase, V0 domain

GO:0031369 19277 50 808565 711 -24.7035 1.86811e-11 GO:translation initiation factor binding

GO:0030117 19277 195 808565 5047 -23.7205 4.99248e-11 GO:membrane coat

GO:0030130 19277 27 808565 245 -23.6638 5.28385e-11 GO:clathrin coat of trans-Golgi network vesicle

GO:0030132 19277 27 808565 245 -23.6638 5.28385e-11 GO:clathrin coat of coated pit

GO:0036459 19277 160 808565 3928 -23.4766 6.37171e-11 GO:thiol-dependent ubiquitinyl hydrolase activity

GO:0006351 19277 432 808565 13564 -21.1784 6.34341e-10 GO:transcription, DNA-templated

GO:0016579 19277 166 808565 4271 -21.0125 7.48824e-10 GO:protein deubiquitination

GO:0006397 19277 48 808565 761 -20.2974 1.53094e-09 GO:mRNA processing

GO:0016757 19277 41 808565 708 -15.4542 1.9423e-07 GO:transferase activity, transferring glycosyl groups

GO:0000151 19277 45 808565 839 -14.6861 4.18716e-07 GO:ubiquitin ligase complex

GO:0034450 19277 45 808565 839 -14.6861 4.18716e-07 GO:ubiquitin-ubiquitin ligase activity

GO:0005852 19277 72 808565 1668 -13.8918 9.26539e-07 GO:eukaryotic translation initiation factor 3 complex

GO:0003774 19277 160 808565 4612 -13.753 1.06452e-06 GO:motor activity

GO:0016459 19277 160 808565 4612 -13.753 1.06452e-06 GO:myosin complex

GO:0004109 19277 28 808565 427 -13.458 1.42984e-06 GO:coproporphyrinogen oxidase activity

GO:0000398 19277 82 808565 2008 -13.4332 1.46572e-06 GO:mRNA splicing, via spliceosome

GO:0016567 19277 48 808565 981 -13.064 2.12014e-06 GO:protein ubiquitination

GO:0005315 19277 35 808565 641 -12.2798 4.64464e-06 GO:inorganic phosphate transmembrane transporter activity

GO:0006817 19277 35 808565 641 -12.2798 4.64464e-06 GO:phosphate ion transport

GO:0000796 19277 36 808565 674 -12.108 5.5153e-06 GO:condensin complex

GO:0007076 19277 36 808565 674 -12.108 5.5153e-06 GO:mitotic chromosome condensation

GO:0000469 19277 15 808565 160 -11.9957 6.17083e-06 GO:cleavage involved in rRNA processing

GO:0008324 19277 26 808565 421 -11.6237 8.95109e-06 GO:cation transmembrane transporter activity

GO:0016021 19277 1331 808565 50487 -11.6022 9.14618e-06 GO:integral component of membrane

GO:0004814 19277 20 808565 282 -11.2405 1.31318e-05 GO:arginine-tRNA ligase activity

GO:0006420 19277 20 808565 282 -11.2405 1.31318e-05 GO:arginyl-tRNA aminoacylation

GO:0006812 19277 44 808565 937 -11.2234 1.33579e-05 GO:cation transport

GO:0006302 19277 18 808565 242 -10.9055 1.83574e-05 GO:double-strand break repair

GO:0016614 19277 18 808565 244 -10.8001 2.03972e-05 GO:oxidoreductase activity, acting on CH-OH group of donors

GO:0031625 19277 59 808565 1425 -10.7186 2.21288e-05 GO:ubiquitin protein ligase binding

GO:0015078 19277 71 808565 1816 -10.6822 2.29491e-05 GO:proton transmembrane transporter activity

GO:0006511 19277 119 808565 3462 -10.5977 2.49745e-05 GO:ubiquitin-dependent protein catabolic process

GO:0001510 19277 16 808565 211 -10.115 4.04685e-05 GO:RNA methylation

GO:0009452 19277 16 808565 211 -10.115 4.04685e-05 GO:7-methylguanosine RNA capping

GO:0005681 19277 30 808565 583 -9.81203 5.47884e-05 GO:spliceosomal complex

GO:0005732 19277 26 808565 477 -9.63271 6.5549e-05 GO:small nucleolar ribonucleoprotein complex

GO:0034457 19277 26 808565 477 -9.63271 6.5549e-05 GO:Mpp10 complex

--------------------------------------------------------------------

homopeptide-depleted GO terms

--------------------------------------------------------------------

GO:0006468 19277 322 808565 20770 -39.9432 4.49677e-18 GO:protein phosphorylation

GO:0004672 19277 322 808565 20746 -39.7339 5.54375e-18 GO:protein kinase activity

GO:0020035 19277 74 808565 6438 -29.2349 2.01116e-13 GO:cytoadherence to microvasculature, mediated by symbiont protein

GO:0005515 19277 194 808565 12894 -28.5021 4.18485e-13 GO:protein binding

GO:0007165 19277 3 808565 1448 -26.063 4.79735e-12 GO:signal transduction

GO:0035556 19277 3 808565 1230 -21.2845 5.70483e-10 GO:intracellular signal transduction

GO:0008654 19277 0 808565 870 -21.0044 7.54964e-10 GO:phospholipid biosynthetic process

GO:0004114 19277 3 808565 1196 -20.5472 1.19255e-09 GO:3',5'-cyclic-nucleotide phosphodiesterase activity

GO:0055114 19277 276 808565 15808 -19.2161 4.51372e-09 GO:oxidation-reduction process

GO:0005975 19277 22 808565 2546 -19.1746 4.70515e-09 GO:carbohydrate metabolic process

GO:0009190 19277 3 808565 1124 -18.994 5.6365e-09 GO:cyclic nucleotide biosynthetic process

GO:0016849 19277 3 808565 1124 -18.994 5.6365e-09 GO:phosphorus-oxygen lyase activity

GO:0019239 19277 0 808565 735 -17.7436 1.96821e-08 GO:deaminase activity

GO:0003700 19277 13 808565 1804 -16.9372 4.40822e-08 GO:DNA-binding transcription factor activity

GO:0003755 19277 16 808565 1951 -16.0131 1.11075e-07 GO:peptidyl-prolyl cis-trans isomerase activity

GO:0030170 19277 9 808565 1453 -15.782 1.39949e-07 GO:pyridoxal phosphate binding

GO:0003723 19277 101 808565 6641 -15.4814 1.89031e-07 GO:RNA binding

GO:0005525 19277 235 808565 13195 -15.0922 2.78964e-07 GO:GTP binding

GO:0004748 19277 0 808565 607 -14.6523 4.33079e-07 GO:ribonucleoside-diphosphate reductase activity, thioredoxin disulfide as acceptor

GO:0016779 19277 0 808565 592 -14.2901 6.22123e-07 GO:nucleotidyltransferase activity

GO:0008234 19277 53 808565 3983 -14.2219 6.66068e-07 GO:cysteine-type peptidase activity

GO:0004743 19277 1 808565 692 -13.8768 9.40548e-07 GO:pyruvate kinase activity

GO:0030955 19277 1 808565 692 -13.8768 9.40548e-07 GO:potassium ion binding

GO:0046983 19277 0 808565 561 -13.5416 1.31514e-06 GO:protein dimerization activity

GO:0000413 19277 16 808565 1777 -13.3089 1.65966e-06 GO:protein peptidyl-prolyl isomerization

GO:0005971 19277 0 808565 536 -12.9379 2.40513e-06 GO:ribonucleoside-diphosphate reductase complex

GO:0009263 19277 0 808565 536 -12.9379 2.40513e-06 GO:deoxyribonucleotide biosynthetic process

GO:0016209 19277 0 808565 525 -12.6723 3.13682e-06 GO:antioxidant activity

GO:0043039 19277 16 808565 1711 -12.3224 4.45098e-06 GO:tRNA aminoacylation

GO:0004512 19277 0 808565 508 -12.2618 4.72885e-06 GO:inositol-3-phosphate synthase activity

GO:0006021 19277 0 808565 508 -12.2618 4.72885e-06 GO:inositol biosynthetic process

GO:0003910 19277 0 808565 497 -11.9962 6.16739e-06 GO:DNA ligase (ATP) activity

GO:0004612 19277 0 808565 469 -11.3202 1.21256e-05 GO:phosphoenolpyruvate carboxykinase (ATP) activity

GO:0016491 19277 123 808565 7199 -11.1972 1.37123e-05 GO:oxidoreductase activity

GO:0006281 19277 14 808565 1506 -11.1338 1.46103e-05 GO:DNA repair

GO:0051536 19277 13 808565 1437 -11.0376 1.60858e-05 GO:iron-sulfur cluster binding

GO:0045454 19277 36 808565 2755 -10.9271 1.79642e-05 GO:cell redox homeostasis

GO:0016747 19277 0 808565 443 -10.6925 2.27156e-05 GO:transferase activity, transferring acyl groups other than amino-acyl groups

GO:0006621 19277 0 808565 441 -10.6442 2.38394e-05 GO:protein retention in ER lumen

GO:0046923 19277 0 808565 441 -10.6442 2.38394e-05 GO:ER retention sequence binding

GO:0006415 19277 6 808565 932 -10.3405 3.22983e-05 GO:translational termination

GO:0000287 19277 25 808565 2072 -10.0598 4.27637e-05 GO:magnesium ion binding

GO:0004809 19277 0 808565 416 -10.0406 4.35934e-05 GO:tRNA (guanine-N2-)-methyltransferase activity
