# Supplementary material for: The relationship between protein domains and homopeptides in the Plasmodium falciparum proteome
Source: PeerJ. 2020 Oct 2;8:e9940. doi: 10.7717/peerj.9940 (PMC7534687; doi:10.7717/peerj.9940)
Supplement: Supplemental Information 3 — The format of this table is modeled on Table 1. [file peerj-08-9940-s003.docx]

**Table S3: Homopeptide enriched/depleted amino-acid types in protein domains, with various degrees of protein domain shortening**

| *Amino Acid (one letter code)* | *Homopeptide amount(≥3) in proteome (N=209236)* | *Homopeptide amount (≥3) in pfam domain (n=19052)* | *P-value ** | *Same, but with 3 residues chopped from domain ends (n=18190)* | *P-value ** | *Same, but with 5 residues chopped from domain ends (n=17761)* | *P-value ** | *Same, but with 7 residues chopped from domain ends (n=17184)* | *P-value ** |
| --- | --- | --- | --- | --- | --- | --- | --- | --- | --- |
| **A** | **954** | **723** | **0** | **717** | **0** | **717** | **0** | **711** | **0** |
| **L** | **8250** | **1676** | **2.44e-222** | **1595** | **3.6e-185** | **1548** | **2e-177** | **1510** | **3e-176** |
| **I** | **7315** | **1527** | **4.69e-214** | **1464** | **2.0e-181** | **1426** | **7e-176** | **1386** | **1.2e-172** |
| **G** | **1527** | **579** | **1.30e-206** | **576** | **1.8e-179** | **561** | **2.3e-174** | **528** | **2.7e-159** |
| **V** | **1108** | **378** | **4.13e-118** | **333** | **1.4e-79** | **312** | **1e-70** | **288** | **2.5e-61** |
| **R** | **1606** | **454** | **1.72e-108** | **435** | **1.7e-89** | **432** | **4.3e-91** | **259** | **4.6e-23** |
| **T** | **2341** | **417** | **2.67e-40** | **357** | **2.6e-24** | **341** | **6.9e-22** | **338** | **2.8e-23** |
| **K** | **48223** | **5036** | **1.81e-31** | **4795** | **2.7e-27** | **4683** | **8.5e-27** | **4585** | **1.8e-30** |
| **S** | **9511** | **1059** | **9.57e-13** | **1033** | **1.9e-14** | **1005** | **8.8e-14** | **984** | **1.1e-14** |
| **P** | **774** | **131** | **2.45e-12** | **122** | **5.8e-10** | **118** | **1.8e-09** | **115** | **1.9e-09** |
| **F** | **3913** | **481** | **4.00e-12** | **453** | **3.5e-09** | **434** | **4.8e-08** | **422** | **4.3e-08** |
| **Y** | **3582** | **393** | **1.5e-05** | **372** | **4.8e-05** | **363** | **5.8e-05** | **357** | **2.3e-05** |
| **C** | **148** | **27** | **0.00022** | **27** | **0.00018** | **27** | **0.00013** | **27** | **8e-05** |
| Q | 1098 | 121 | 0.00384 | 121 | **0.00121** | 118 | **0.00137** | 115 | **0.00126** |
| E | 12572 | 1170 | 0.00914 | 1043 | 0.004 | 974 | **0.00018** | 924 | **3e-05** |
| W | 9 | 3 | 0.03576 | 3 | **0.0001** | 3 | **0.00009** | 3 | **0.00009** |
| M | 189 | 22 | 0.04567 | 22 | 0.035 | 22 | 0.031 | 22 | 0.025 |
| **N** | **92722** | **3941** | **0** | **3820** | **0** | **3794** | **0** | **3749** | **0** |
| **D** | **12636** | **881** | **7.83e-20** | **869** | **8.5e-14** | **850** | **2.3e-13** | **828** | **2e-12** |
| **H** | **758** | **33** | **2.31e-07** | **33** | **3.5e-06** | **33** | **7.5e-06** | **33** | **2e-05** |

*** P-value threshold = 0.0025 (with a Bonferroni correction accounting for tests on the twenty amino acids). P-values of 0.0 are infinitesimally small beyond the precision of the computation. Significant enrichments are in bold and significant depletions in bold underlined. P-values that become significant for different numbers of residues chopped from domain ends are in green bold.**
