# Supplementary material for: The relationship between protein domains and homopeptides in the Plasmodium falciparum proteome
Source: PeerJ. 2020 Oct 2;8:e9940. doi: 10.7717/peerj.9940 (PMC7534687; doi:10.7717/peerj.9940)
Supplement: Supplemental Information 4 — The format of this table is modeled on Table 1. [file peerj-08-9940-s004.docx]

| **Table S4: Homopeptide enriched/depleted amino-acid types in protein domains in other *Plasmodium* proteomes*** | | | | | | | | | | | | |
| --- | --- | --- | --- | --- | --- | --- | --- | --- | --- | --- | --- | --- |
| *Amino Acid (one letter code)* | *(1)*  *Homopeptide amount(≥3) in proteome (N=209236)* | *(2)*  *Homopeptide amount (≥3) in pfam domain (n=19052) in* ***Pf strain 3D7*** | *P-value ** | *Same as (1), but for* ***Pf strain FCH/4***  *(N=188195)* | *Same as (2), but for* ***Pf strain FCH/4***  *(n=15208)* | *P-value ** | *Same as (1), but for* ***P. yoelii strain 17XNL***  *(N=109329)* | *Same as (2), but for* ***P. yoelii strain 17XNL***  *(n=17247)* | *P-value ** | *Same as (1), but for* ***P. vivax strain Salvador I***  *(N=106886)* | *Same as (2), but for* ***P. vivax strain Salvador I***  *(n=13815)* | *P-value ** |
| **A** | **954** | **723** | **0** | **744** | **527** | **0** | **717** | **240** | **1.8e-25** | **4617** | **545** | **0.002** |
| **L** | **8250** | **1676** | **2.44e-222** | **7338** | **1484** | **0** | **5789** | **1529** | **2.4e-82** | **9415** | **1874** | **1.2e-76** |
| **I** | **7315** | **1527** | **4.69e-214** | **6812** | **1291** | **1.4e-178** | **6974** | **2130** | **1.2e-179** | **2436** | **813** | **4.3e-126** |
| **G** | **1527** | **579** | **1.30e-206** | **1258** | **511** | **5e-185** | **1403** | **361** | **4e-18** | **13248** | **972** | **1.3e-94** |
| **V** | **1108** | **378** | **4.13e-118** | **848** | **287** | **8e-87** | **859** | **328** | **1.1e-43** | **1607** | **499** | **0** |
| **R** | **1606** | **454** | **1.72e-108** | **1455** | **337** | **3e-62** | **1051** | **127** | **2.6e-04** | **4397** | **329** | **4.8e-29** |
| **T** | **2341** | **417** | **2.67e-40** | **2202** | **382** | **7e-39** | **1329** | **365** | **5e-24** | **1537** | **348** | **1.2e-24** |
| **K** | **48223** | **5036** | **1.81e-31** | **40851** | **3796** | **8e-23** | **31391** | **3831** | **6.3e-84** | **24694** | **3376** | **8.2e-06** |
| **S** | **9511** | **1059** | **9.57e-13** | **8590** | **808** | **3.2e-06** | **8454** | **3029** | **0** | **10889** | **1173** | **0** |
| **P** | **774** | **131** | **2.45e-12** | **666** | **100** | **3.1e-09** | 824 | 145 | 0.013 | **3333** | **245** | **1.5e-23** |
| **F** | **3913** | **481** | **4.00e-12** | **3659** | **394** | **5.1e-10** | **3213** | **352** | **2.5e-13** | 3340 | 452 | 0.01 |
| **Y** | **3582** | **393** | **1.5e-05** | **3218** | **303** | **5.8e-04** | **2038** | **240** | **3.1e-07** | **1284** | **258** | **5.7e-12** |
| **C** | **148** | **27** | **0.00022** | **122** | **24** | **1.7e-04** | 49 | 15 | 0.006 | **126** | **36** | **1.3e-05** |
| Q | 1098 | 121 | 0.00384 | 833 | 71 | 0.042 | **869** | **33** | **6.4e-12** | **1136** | **80** | **5.4e-10** |
| E | 12572 | 1170 | 0.00914 | 10218 | **881** | **0.0015** | **7036** | **859** | **3.7e-16** | **15139** | **1655** | **2e-15** |
| W | 9 | 3 | 0.03576 | 6 | 3 | 0.012 | 3 | 0 | 0.62 | 9 | 3 | 0.08 |
| M | 189 | 22 | 0.04567 | 212 | 19 | 0.08 | 77 | 19 | 0.02 | 204 | 38 | 0.006 |
| **N** | **92722** | **3941** | **0** | **88285** | **3311** | **0** | **32032** | **2853** | **0** | 4307 | 612 | 0.001 |
| **D** | **12636** | **881** | **7.83e-20** | **10229** | **654** | **6.7e-11** | 4949 | 761 | 0.01 | **4525** | **471** | **1.33e-05** |
| **H** | **758** | **33** | **2.31e-07** | **649** | **25** | **1.5e-05** | 225 | 30 | 0.04 | **633** | **30** | **3e-11** |

*** P-value threshold = 0.0025 (with a Bonferroni correction accounting for tests on the twenty amino acids). P-values of 0.0 are infinitesimally small beyond the precision of the computation. Significant enrichment in domains are in bold, and significant depletions in bold underlined. P-values that become significant for different Plasmodium species/strains are in green bold, and those that lose significance in red.**
